# A Pancreatic Head Tumor Arising as a Duodenal GIST: A Case Report and Review of the Literature

**DOI:** 10.1155/2014/420295

**Published:** 2014-09-10

**Authors:** Fabian Bormann, Wolfgang Wild, Hüseyin Aksoy, Pius Dörr, Sanja Schmeck, Matthias Schwarzbach

**Affiliations:** ^1^Klinik für Allgemein-, Viszeral-, Thorax- und Gefäßchirurgie, Klinikum Frankfurt Höchst, Gotenstraße 6-8, 65929 Frankfurt am Main, Germany; ^2^Klinik für Radiologie, Neuroradiologie und Nuklearmedizin, Klinikum Frankfurt Höchst, Gotenstraße 6-8, 65929 Frankfurt am Main, Germany; ^3^Institut für Pathologie, Klinikum Frankfurt Höchst, Gotenstraße 6-8, 65929 Frankfurt am Main, Germany

## Abstract

Gastrointestinal stromal tumors (GISTs) are rare mesenchymal tumors of the gastrointestinal tract that originate from the *intestinal cells of Cajal* (ICC) (Fletcher et al., 2002). Only a few cases have been described with extragastrointestinal stromal tumors (Kim et al., 2012; Soufi et al., 2013; Meng et al., 2011). They are often diagnosed as a pancreatic head tumor as they are very difficult to relate to the duodenum with CT, MRI, or ultrasound. We present a case of a sixty-four-year-old woman who presented with abdominal pain and cardialgia for a follow-up examination after breast cancer surgery. On laparotomy there was a 3 × 5 cm hypervascular mass arising from the pancreatic head with macroscopically no attachment to the duodenum. The patient underwent pancreatoduodenectomy (PD) modified after Traverso-Longmire, histopathology proved a duodenal GIST. This case proves that duodenal GISTs can grow invasively into the pancreas and appear as solid pancreas head tumor; therefore, these tumors should be included into differential diagnosis.

## 1. Introduction

GISTs are the most common mesenchymal tumors of the GI [1]. They may arise in any part of the GI but mostly occur in the stomach (60%) and the small intestine (35%). Only a few cases have been described with extragastrointestinal stromal tumors [2–4]. They are a very rare tumor identity among the duodenal tumors with less than 5%.

## 2. Case Report

A sixty-four-year-old woman, in good physical condition, with abdominal pain and cardialgia for four weeks was transferred to our clinic. She first presented in the gynecological department for her follow-up examination after a surgical breast cancer treatment nine years ago. The outwards ultrasound of the liver and the upper abdomen showed an unknown formation in the pancreas. The result of the followed endoscopic biopsy showed a carcinoid with an unknown dignity.

**Figure 1:**
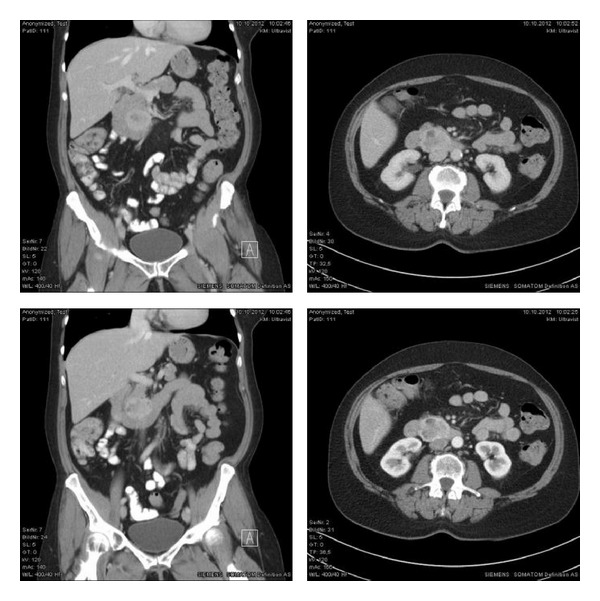
CT-scan of the abdomen with the pancreatic mass of enhancing and nonenhancing areas.

In the anamnesis, she described a slowly increasing pain within the last few days, mostly located in the right upper abdomen. She could not tell any relief or aggravation or any kind of radiation. She was not suffering from fever, night sweat, vomiting, jaundice, or weight loss. The history of bowel movement was empty. The physical examination showed a soft abdomen, without any resistance. The pain slightly increased by pressure in the epigastric area. The gallbladder could not be palpated and the liver margin seemed to be inconspicuous. There was no abnormality on digital rectal examination. The routine laboratory tests showed no pathological findings. In our abdominal ultrasound, we found the suspicious heterogeneous formation of 3 × 5 cm in the pancreas and a 3 × 1 cm cystic formation in liver segment 6. The CT-scan proved the ultrasound findings and showed the described formation in the pancreatic head, and a connection to the duodenum could not be found. The well-defined mass of heterogeneous density seemed to consist of enhancing and nonenhancing areas (Figure 1).

In further staging examinations, there was no pathological finding for the tumor markers* CEA*,* Ca-19 9*,* gastrin*, and* chromogranin*. A gastroscopy showed a moderate streaky gastritis of the antrum without any ulcer. The especially for neuroendocrine tumors very sensitive 99mTc-EDDA/HYNIC-TOC scintigraphy did not show any strong enhancement. The tumor could easily be seen by exploratory laparotomy as a hypervascular mass arising from the pancreatic head, which was mobile to the retroperitoneum and seemed to be resectable. It did not infiltrate into the pylorus and macroscopically it did not attach to the duodenum. There was also no peritoneal dissemination. The suspicious liver area was localized and marked by ultrasound. PD modified after Traverso-Longmire was performed and the marked liver area in segment 6 was resected in liver wedge technique.

Histopathology showed a mesenchymal, sharply margined tumor of 4 cm size, consisting of spindle cells and isolated apoptosis but without necrosis. Mitosis could not be detected. On one side of the tumor, a small connection to the duodenal serosa was conceivable.

**Figure 2:**
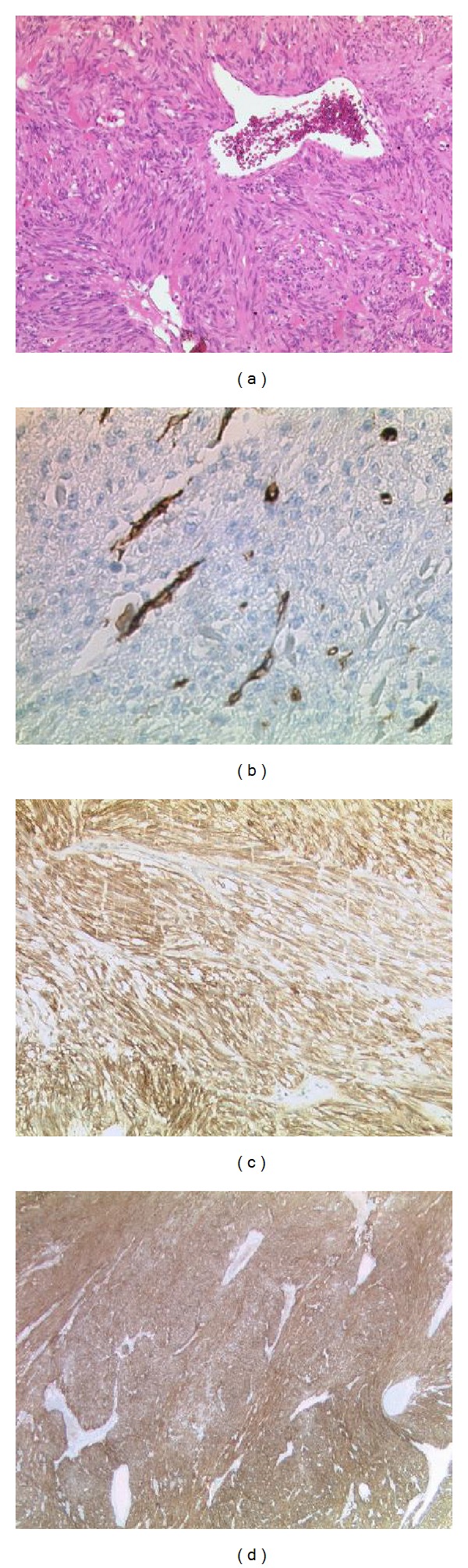
Histopathological pictures of different stainings. (a) Hematoxylin-eosin staining. (b) CD 34 staining. (c) CD117 staining. (d) DOG-1 staining.

In the immunohistology, the tumor was positive for* smooth muscle actin* (*ASMA*),* bcl-2*,* CD 99*,* CD 117*,* DOG-1*, and some cells also for* CD 34* (Figure 2). Negative staining for* desmin* and* S-100*. *MIB-1* a marker for proliferation showed a rate of 1%. Further examinations showed an exon 11 mutation in the c-KIT gene.

Based on these findings, the tumor was finally diagnosed as a low risk GIST arising from the duodenal wall, incorporating into the pancreatic head.

Postoperatively the patient suffered from a pneumonia, which was treated with antibiotics. There was no raise of amylase and lipase in the drainage; it was removed at day ten after surgery. The blood glucose was not elevated. The patient was discharged in good condition and advised to take pancreatin beside the meals.

## 3. Discussion

GISTs are quite rare tumors with an incidence of annually only 10–15 per million [1]. They are the most common mesenchymal tumors of the GI, originating from the ICC, a cell from the autonomic nervous system. For sporadic GISTs, there is no gender difference and the median age at the time of diagnosis is 60 years.

GISTs can occur anywhere in the GI but mostly affect the stomach (60%), jejunum and ileum (30%) only 4-5% arise in den duodenum. Depending on the size and location of the tumor, they often present with abdominal discomfort, full feeling, and gastrointestinal bleeding, if the tumor grows invasively into the epithelial layer. At time of diagnosis, approximately 20–50 % of the patients already have metastasis, most likely in the liver and/or the peritoneum. Very rarely can metastasis be found in the lymph nodes; therefore, surgery does not have to be that radical [5, 6].

The endoscopy that is often performed by the described symptoms above frequently shows a submucosal bulge or ulceration. Together with an endoscopic ultrasound, the origin can be displayed intra- or extramurally. However, the final diagnosis is proofed by biopsy [5, 7, 8], which is often performed in a CT or ultrasound guided way. The percutaneous biopsy should only be considered if another differential diagnosis (e.g., lymphoma) or a neoadjuvant treatment is likely. As GISTs are quite fragile and well perfused, it is difficult to perform the biopsy without spreading the tumor cells into the abdominal cavity. With an intraabdominal rupture of the tumor capsule, the development of peritoneal metastasis is almost obligatory. In case of doubt, the tumor should be primarily resected.

GISTs typically express the* c-KIT* protein (*CD 117*). Histopathologically the main mechanism in the genesis of a GIST is the mutation in one of two receptor tyrosine kinase genes. Most frequently the mutation is located on KIT exon 11 (70%), KIT exon 9 (10%), and PDGFRA exon 18 (5%). GISTs mostly occur in a spindle cell type (60–70%) but may also occur in an epithelioid type (20–30%) or as a mixture. Other typical markers for GISTs are the antibody* Discovered on GIST-1* (*DOG-1*), the cell surface glycoprotein* CD34* (70% are positive), and* ASMA* (40% are positive). GISTs are mostly negative for* desmin* and* S-100* [5, 9, 10].

If resectable, the tumor should be treated by surgery. Depending on the localization and the size of the tumor, this can be performed laparoscopically or by conventional laparotomy. To be considered as potentially curative, the resection needs to be with a tumor free margin and a safety clearance of at least 1 cm [7, 8].

As in our case described herein, most duodenal GISTs in the pancreas first seem to be a pancreatic head tumor of neuroendocrine origin. In these cases, the surgical resection with tumor free margin is desirable; therefore, we decided to treat with a PD. As there is no benefit in survival for patients with a duodenal GIST treated with PD compared to limited resection (LR), it is very important to optimize the diagnosis to minimize the surgical extent and the morbidity [11, 12].

If the tumor is primarily not resectable (size or location) or already with a metastatic spread neoadjuvant chemotherapy for example, with imatinib should be performed. Within 3-4 months, the tumor should be reevaluated and secondly resected [7, 13, 14].

Regarding the prognosis of malignancy, Miettinen et al. classified by the amount of mitosis per 50 HPF the size and origin of the tumor [15] (Table 1).

Fletcher classified in a similar way [1]. Patients with duodenal GITSs classified as intermediate or high risk for a tumor relapse should be treated with imatinib 400 mg daily for three years. There is no benefit for patients classified at low risk or with a certain PDGFRA mutation (PDGFRA-D842V) [16–18].

The median 5-year survival of resected patient without metastasis is around 50% without further chemotherapy or biologicals [15, 19–21].

After complete tumor resection, the follow-up care should be within every 3–6 months, including clinical examination and CT-scans of the abdomen and the pelvis once a year for 5 years [16].

## Figures and Tables

**Table 1:** Risk classification of duodenal GISTs modified after Miettinen and Lasota [15].

Amount of mitosis	Size (cm)	Risk
≤5 per 50 HPF	≤2	No risk
	2–5	Low
	5–10	High

>5 per 50 HPF	≤2	No data
	2–5	High
	5–10	High
